# Biomarkers for differential diagnosis of myocarditis, acute coronary syndrome, and sepsis-induced cardiomyopathy

**DOI:** 10.3389/fcvm.2026.1956085

**Published:** 2026-10-26

**Authors:** Yuanyuan Xiao, Tao Xiang

**Affiliations:** Department of Emergency, The Third People’s Hospital of Chengdu, Chengdu, Sichuan, China

**Keywords:** acute coronary syndrome, acute myocardial injury, biomarkers, differential diagnosis, microRNA, myocarditis, sepsis-induced cardiomyopathy

## Abstract

**Backgrounds:**

Diagnosing acute myocardial injury remains a significant challenge in contemporary cardiovascular and critical care medicine. Myocarditis, acute coronary syndrome (ACS), and sepsis-induced cardiomyopathy (SICM) frequently present with overlapping features, chest pain, elevated cardiac biomarkers, and electrocardiographic abnormalities, yet require divergent management, making rapid and accurate etiological diagnosis essential for optimal patient outcomes.

**Methods:**

This narrative review synthesizes current evidence on the pathophysiology and biomarker signatures of myocarditis, ACS, and SICM, and examines how these signatures can be integrated into a practical diagnostic approach for the emergency department and intensive care unit.

**Results:**

High-sensitivity cardiac troponin (hs-cTn) remains the central biomarker of acute myocardial injury, but its kinetics differ across the three conditions. A steep rise and fall in ACS, a sustained moderate elevation in myocarditis, and a gradual, severity-parallel course in sepsis. Among complementary biomarkers, C-reactive protein lacks specificity, whereas procalcitonin supports the recognition of bacterial sepsis in SICM, and natriuretic peptides reflect myocardial wall stress, disproportionately elevated relative to troponin in SICM. Emerging molecular biomarkers, circulating microRNAs (like miR-4763-3p and miR-155) and extracellular matrix remodeling markers, show promise for distinguishing inflammatory from ischemic myocardial injury, but remain investigational owing to the lack of standardized assays and practical constraints on acute bedside use. Therefore diagnosis rests on an integrated, multimodal assessment.

**Conclusion:**

The differential diagnosis of acute myocardial injury requires a systematic, biomarker-centered multimodal approach. Future research should prioritize the prospective validation of multi-biomarker panels and the development of rapid non-invasive tools to reduce reliance on emergency coronary angiography.

## 1 Introduction

Diagnosing acute myocardial injury remains a significant challenge in contemporary cardiovascular medicine. Myocarditis, acute coronary syndrome (ACS), and sepsis-induced cardiomyopathy (SICM) frequently present with overlapping features, such as chest pain, elevated cardiac biomarkers, and electrocardiographic abnormalities, yet require divergent management strategies, making rapid and accurate etiological diagnosis essential for optimal outcomes.

The inflammatory myopericardial syndrome (IMPS), spaning a continuous spectrum from isolated pericarditis to isolated myocarditis, encompassing two overlapping phenotypes, myopericarditis and perimyocarditis (1). Acute myocarditis, in particular, often mimics acute myocardial infarction(AMI), with chest pain, ST-segment elevation, malignant arrhythmias, and reduced left ventricular ejection fraction (LVEF) (2). Because the two conditions cannot be reliably distinguished by symptoms, ECG, or routine laboratory markers alone, the European Society of Cardiology (ESC) assigns a class IC recommendation to coronary angiography to rule out ACS in patients with suspected myocarditis (1). This requirement is clinically burdensome. Catheterization facilities with an on-call physician are often unavailable, necessitating inter-hospital transfer under emergency care support (3, 4), and angiography is an invasive procedure carrying a 1%–2% risk of serious complications, including contrast reactions and contrast-induced nephropathy (5). These difficulties are compounded by shifting demographics, as myocardial infarction(MI) increasingly affects younger patients, in whom myocarditis and ACS may be particularly difficult to separate at the bedside (6), it can still be hard to tell them apart quickly in the emergency department, especially as more young people experience heart attacks. Consequently, a rapid, non-invasive means of discriminating MI from myocarditis remains an unmet clinical need (7, 8). The true rate of myocarditis in the population is not well known (9), since the gold standard test, endomyocardial biopsy (EMB), is rarely done in practice (8). Estimates suggest that about 22 people per 100,000 worldwide get myocarditis each year (10). It remains a poorly understood inflammatory disease with many possible causes, including infections, systemic diseases, drugs, and toxins (11).

Each year, approximately 15 million patients in Europe and the United States present to the emergency department with chest pain or other symptoms suggestive of AMI (12). Time is critical, as delayed reperfusion in ST-elevation myocardial infarction (STEMI) increases mortality. Current ESC guidelines mandate an immediate reperfusion strategy, preferably primary coronary angiography, with a maximum system delay of 120 min from STEMI diagnosis (13). Accordingly, only after obstructive coronary artery disease has been excluded may alternative diagnoses be pursued and adjunctive imaging, such as echocardiography and cardiac magnetic resonance (CMR), be undertaken (14). The recently published 2025 ESC Guidelines for the management of myocarditis and pericarditis reaffirm the role of coronary angiography, alongside EMB, in patients with clinically suspected myocarditis (1).

SICM adds a third dimension to this differential diagnosis. Sepsis is defined as life-threatening organ dysfunction caused by a dysregulated host response to infection (15). Cardiac compromise ranks among the most serious sequelae of this syndrome (16). Troponin elevation is common in septic patients and arises largely through non-ischemic mechanisms, including increased membrane permeability, microcirculatory dysfunction, and catecholamine toxicity (17–19). However, controversy persists over whether troponin elevation, or its maximal value, establishing the diagnosis of SICM (20–22). As a clinical entity, SICM remains poorly characterized and complex, exerting considerable influence on management strategies and patient outcomes in sepsis and septic shock. The confluence of nonstandardized diagnostic frameworks and contradictory evidence regarding both echocardiography and biomarker application severely constrains accurate identification and clinical management (23).

SICM needs to be differentiated from other causes of acute cardiac dysfunction, mainly ACS, which needs to be rapidly and promptly diagnosed and treated. Myocarditis and SICM may also be difficult to differentiate, as they both appear in patients with infections. But generally, myocarditis is associated with viral infections whereas SICM has been described in bacterial sepsis (24). Because these three conditions can look similar in cases of acute myocardial injury, it is important to use a systematic approach that combines clinical evaluation, biomarker analysis, electrocardiography, and, when needed, invasive tissue sampling. This review provides, to our knowledge, the first integrated biomarker-based framework for the differential diagnosis of acute myocardial injury across these three conditions, with emphasis on emerging markers and bedside triage tools.

## 2 Pathophysiological overview

### 2.1 Myocarditis

Acute myocarditis is an inflammatory condition that affects the heart muscle, most often triggered by viruses (25). Some viruses, like enteroviruses and adenoviruses, directly damage heart cells. Others, such as parvovirus B19 and human herpesvirus 6, cause harm by activating the body's immune response (8, 26–30). The disease usually starts with viral damage to the heart and an early immune reaction. This is followed by a phase in which CD3+ T-cells and CD68+ macrophages are the main immune cells involved, and autoantibodies are sometimes produced (1, 31). The inflammation can become more severe through the activation of the NLRP3 inflammasome, which leads to the release of interleukin-1β and interleukin-18 (32, 33). People with certain genetic variants, especially in desmosomal and sarcomeric genes, may be more likely to have strong inflammatory responses (34–36).

### 2.2 Acute coronary syndrome

ACS comprises a spectrum of acute myocardial ischemia, ranging from unstable angina (transient ischemia without necrosis) to STEMI (transmural infarction), which arises when an unstable atherosclerotic plaque undergoes erosion, fissuring, or rupture, triggering intraluminal thrombosis and compromised coronary blood flow. The clinical phenotype is determined by the extent and duration of obstruction (37–39). Plaque vulnerability is driven by sustained arterial wall inflammation. Macrophages within the necrotic core secrete matrix metalloproteinases (MMPs) that degrade the fibrous cap, while pro-inflammatory signaling upregulates tissue factor, creating a pro-thrombotic milieu that predisposes to rupture (40).

### 2.3 Sepsis-induced cardiomyopathy

SICM is a reversible myocardial dysfunction syndrome occurring in 30%–60% of patients with severe sepsis or septic shock, depending on the diagnostic criteria and population studied (15, 41). Its pathophysiology is multifactorial. Circulating pathogen- and damage-associated molecular patterns activate Toll-like receptor 4 (TLR-4), driving the release of inflammatory mediators, particularly tumor necrosis factor-α (TNF-α) and interleukin-1β (IL-1β), which depress cardiomyocyte contractility through nitric oxide (NO)-mediated pathways and calcium desensitization (42–45). Concurrent peripheral vasodilation, microvascular thrombosis (46, 47), and mitochondrial dysfunction (48) with excessive reactive oxygen species (ROS) production further impair cellular energy metabolism and promote myocardial depression (49, 50). Figure 1 provides an integrated overview of the pathophysiological mechanisms, the shared inflammatory cascade, and the complementary biomarker panel discussed in the following sections.

**Figure 1:**
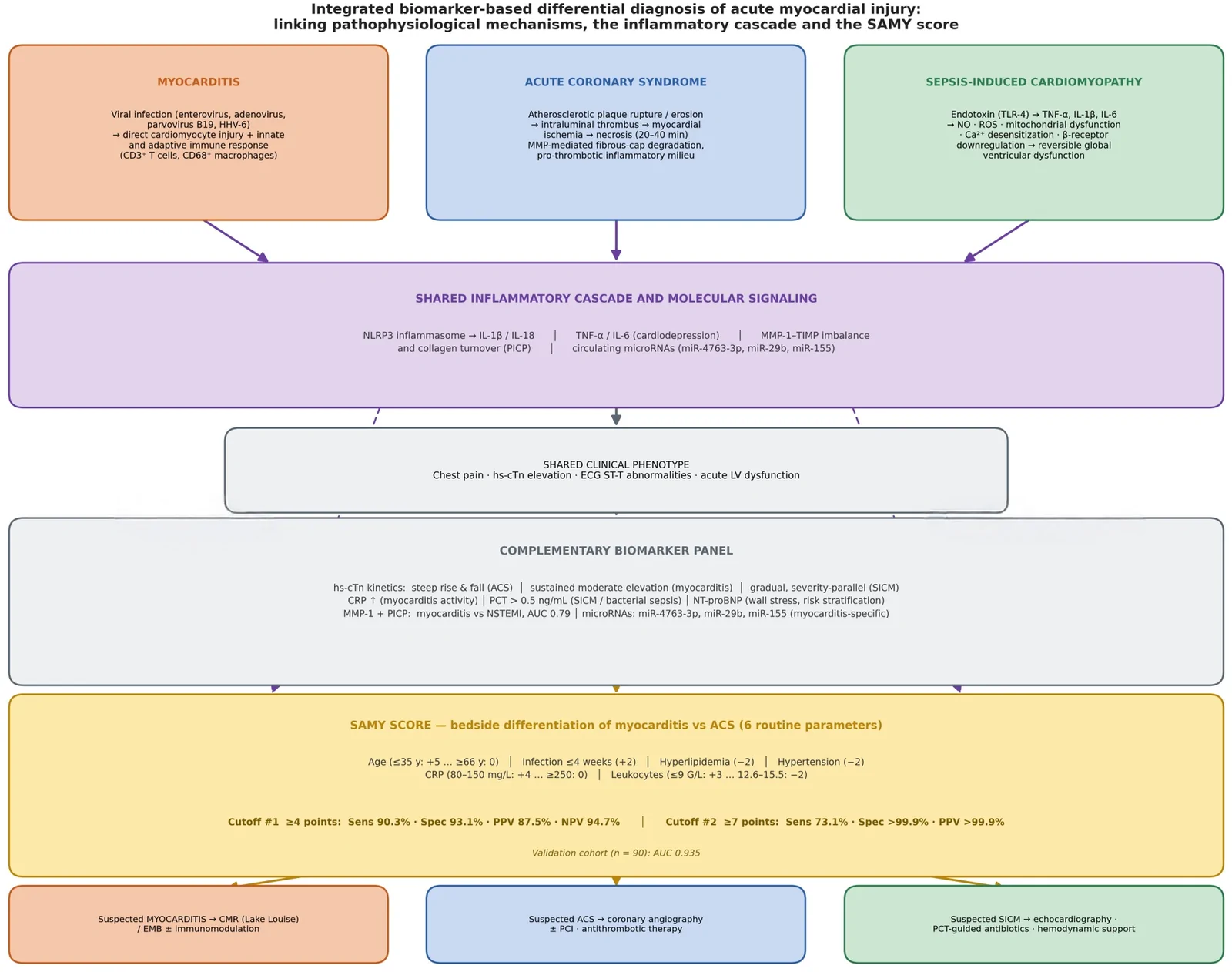
Integrated schematic linking the pathophysiological mechanisms of myocarditis, ACS and SICM to the shared inflammatory cascade, complementary biomarker panel and the clinical components of the SAMY score, guiding subsequent diagnostic pathways (CMR/EMB vs. coronary angiography vs. sepsis-focused management). ACS, acute coronary syndrome; AUC, area under the curve; CMR, cardiac magnetic resonance; CRP, C-reactive protein; ECG, electrocardiogram; EMB, endomyocardial biopsy; HHV-6, human herpesvirus 6; hs-cTn, high-sensitivity cardiac troponin; IL, interleukin; LV, left ventricular; MMP-1, matrix metalloproteinase-1; miRNA, microRNA; NPV, negative predictive value; NSTEMI, non-ST-elevation myocardial infarction; NT-proBNP, N-terminal pro-B-type natriuretic peptide; NO, nitric oxide; PCI, percutaneous coronary intervention; PCT, procalcitonin; PICP, procollagen type I carboxy-terminal propeptide; PPV, positive predictive value; ROS, reactive oxygen species; SAMY, SAlzburg MYocarditis; Sens, sensitivity; SICM, sepsis-induced cardiomyopathy; Spec, specificity; TIMP, tissue inhibitor of metalloproteinases; TLR-4, Toll-like receptor 4; TNF-α, tumor necrosis factor-alpha.

## 3 Cardiac troponin: the central biomaker

Hs-cTn assays have transformed the diagnosis of acute myocardial injury across all three conditions, detecting circulating troponin at concentrations 10–100 fold lower than conventional assays (51, 52). This sensitivity comes at the cost of specificity, as numerous non-ischemic conditions produce detectable elevations. Serial measurements using 0/1 h or 0/2 h algorithms are therefore essential, since absolute delta changes distinguish acute from chronic injury (39). However, substantial delta changes can also occur in myocarditis and SICM, and little or no change may be seen when sampling near the cTn peak in ACS (37, 53). The differential kinetic patterns of hs-cTn across the three conditions are summarized in Table 1.

**Table 1:** High-sensitivity cardiac troponin (hs-ctn) kinetics across myocarditis, acute coronary syndrome (ACS) and sepsis-induced cardiomyopathy (SICM).

Feature	Myocarditis	ACS (STEMI/NSTEMI)	SICM
Peak levels	Moderate; generally lower than in transmural infarction (considerable overlap)	High (>5× the 99th percentile URL in STEMI); more modest in NSTEMI	Typically mild to moderate
Kinetic pattern	Gradual rise; sustained, prolonged elevation	Steep rise and fall; early peak (12–24 h) with subsequent decline	Gradual rise paralleling disease severity; decline with clinical recovery
Release mechanism	Inflammatory cell–mediated myocyte membrane disruption	Ischemic necrosis (plaque rupture with superimposed thrombosis)	Cytokine-induced membrane permeability; microcirculatory dysfunction;

ACS, acute coronary syndrome; AMI, acute myocardial infarction; hs-cTn, high-sensitivity cardiac troponin; NSTEMI, non-ST-elevation myocardial infarction; SICM, sepsis-induced cardiomyopathy; STEMI, ST-elevation myocardial infarction; URL, 99th percentile upper reference limit.

### 3.1 cTn elevation patterns in myocarditis

In acute myocarditis, cTn is elevated in approximately one third to nearly all patients, depending on disease severity and when the measurement is taken (26, 54). The characteristic pattern is a sustained, moderate increase, a slower rise and more prolonged elevation than in STEMI, reflecting inflammatory cell-mediated myocyte membrane disruption rather than ischemic necrosis (26). Peak levels are generally lower than in transmural infarction, although considerable overlap exists.

### 3.2 cTn in ACS

In ACS, hs-cTn demonstrates a steep rise (>5× the sex-specific 99th percentile upper reference limit in STEMI) with an early peak (12–24 h) and subsequent gradual decline. NSTEMI produces more modest elevations with variable kinetics depending on the extent of ischemic injury (37, 39, 55).

### 3.3 cTn in sepsis-induced cardiomyopathy

Elevations are observed in approximately 40%–90% of septic patients, depending on the population studied, and are associated with left ventricular systolic, right ventricular and left ventricular diastolic dysfunction (18, 19, 56, 57). Elevation is typically modest, rises gradually in parallel with disease severity, and falls with clinical recovery, reflecting cytokine-induced membrane permeability and microcirculatory dysfunction rather than established necrosis (42, 48), consistent with a functional and potentially reversible process. The magnitude of elevation carries graded prognostic significance (18, 56, 58). Serial measurements may be informative during follow-up, and persistent or recurrent elevation warrants reassessment for complications (18).

## 4 Complementary biomarkers for differential diagnosis

### 4.1 Established complementary biomarkers

#### 4.1.1 Inflammatory markers

C-reactive protein (CRP), an IL-6-driven acute-phase reactant, rises in parallel with myocardial inflammation and therefore reflects disease activity in myocarditis, however, it lacks specificity, as elevations also occur in ACS (secondary to plaque inflammation), pulmonary embolism, and various systemic conditions (59). Procalcitonin (PCT), by contrast, exhibits superior specificity for bacterial infection: levels typically exceed 0.5 ng/mL in sepsis and correlate closely with disease severity (60), making PCT the key inflammatory marker for distinguishing SICM from viral myocarditis or uncomplicated ACS (24, 61). IL-6 is markedly elevated in severe sepsis and in cytokine storm syndromes, including fulminant myocarditis (62). However, its measurement remains confined to research settings and is not routinely available.

#### 4.1.2 Natriuretic peptides

Natriuretic peptides are frequently elevated in sepsis, in part because pro-inflammatory interleukins stimulate their synthesis within the cardiac chambers in response to myocardial dysfunction (48, 63, 64). This elevation carries differential-diagnostic value, a disproportionate rise in NT-proBNP relative to cardiac troponin suggests that ventricular dysfunction, rather than extensive myocyte necrosis, is the dominant process, thereby supporting a diagnosis of SICM (63). Beyond their diagnostic adjunct, natriuretic peptides also hold prognostic significance in sepsis, as higher levels have been associated with disease severity and adverse outcomes, although they remain supportive rather than definitive markers of SICM (65–67). In myocarditis, markedly elevated NT-proBNP in the absence of obstructive coronary disease should raise suspicion of fulminant disease requiring urgent intervention (26).

### 4.2 Extracellular matrix remodeling markers

MMPs are key regulatory molecules for extracellular matrix degradation (68). MMP-1 is a highly upregulated matrix metalloproteinase in the early stages of myocardial injury, playing a key role in promoting cell migration, controlling tissue structure, and balancing pro-inflammatory and anti-inflammatory stimuli (69, 70). The resultant activity and abundance of MMP-1 are thus dictated by the substrate landscape, temporal-spatial limitations, and the encompassing inflammatory context (68). Procollagen type I carboxy-terminal propeptide (PICP) circulates as the residual C-terminal fragment liberated when procollagen type I is processed into mature, fibril-forming collagen (71). Its serum levels correspond to the myocardial collagen volume fraction on histology (72), and have been connected to worse prognosis across a spectrum of cardiac diseases (73, 74). A study demonstrated that MMP-1 and PICP measurements may differentiate infarct-like myocarditis from NSTEMI in individuals with chest pain, elevated troponins, and non-ST-elevation ECG findings (75). Although such data support the feasibility of using these biomarkers to avert invasive procedures and streamline diagnostic evaluation, prospective validation in external emergency department cohorts is essential (75).

### 4.3 Novel biomarkers

#### 4.3.1 Circulating MicroRNAs

MicroRNAs (miRNAs) are stable, short, single-stranded, non-coding RNAs found in the body. They may be useful as diagnostic and prognostic biomarkers for coronary artery disease. Research shows that miRNAs play important roles in the development of several heart conditions, such as MI, arrhythmias, heart failure, and coronary heart disease (76). Studies suggest that miRNAs could help doctors distinguish between patients with myocarditis or Takotsubo syndrome and those with AMI. They may also help predict outcomes in patients with ACS and detect changes in the left ventricle after an ischemic event (77).

##### 4.3.1.1 MiR-4763-3p

Nie et al. found that miR-4763-3p levels were much higher only in patients with fulminant myocarditis, while miR-4281 was increased in both myocarditis and MI groups compared to healthy people (78). Interestingly, higher miRNA levels were linked to less severe disease in fulminant myocarditis, which suggests miR-4763-3p might help regulate the disease. These results show that miR-4763-3p could be a specific biomarker to tell myocarditis apart from MI.

##### 4.3.1.2 MiR-29b and miR-125b

Patients with fulminant myocarditis had higher levels of miR-29b and miR-125b in their blood, which were positively correlated with the extent of myocardial edema and negatively correlated with left ventricular function. MiR-29b was better than miR-125b at diagnosing fulminant myocarditis (79). These miRNAs have not been reported as discriminatory markers in the context of MI.

##### 4.3.1.3 MiR-155

MiR-155 levels were higher in several groups of myocarditis patients, including those with viral and fulminant types, and were linked to more inflammatory cells in the heart (80, 81). However, miR-155 is not as useful for diagnosing AMI, which suggests it may be more specific for inflammation than for ischemia. In summary, many studies have looked at how miRNAs are involved in myocarditis, but most of this research has been done in the lab rather than with patients. Importantly, miRNA patterns in myocarditis patients differ from those in people with other heart conditions like MI or non-inflammatory dilated cardiomyopathy (DCM) (82). This distinction makes miRNAs promising biomarkers for the diagnosis of myocarditis, however, translation into routine practice awaits standardized assays, validated cut-off values and prospective multicentric validation.

The biomarkers discussed in Section 4 differ substantially in their readiness for clinical use. Hs-cTn, CRP, PCT and natriuretic peptides are established, standardized and immediately actionable at the bedside. By contrast, miRNA panels and the MMP-1/PICP axis remain investigational. Evidence derives predominantly from small, single-center, retrospective cohorts, and no internationally standardized assays or validated decision thresholds currently exist.

## 5 Diagnostic algorithms and scoring systems

### 5.1 The SAlzburg MYocarditis (SAMY) score

The SAMY score is a proven clinical tool that helps distinguish acute myocarditis from ACS in patients with chest pain. Mirna and colleagues (6) developed and validated this score, which uses six easily accessible parameters, as shown in Table 2. The SAMY score's principal strength lies in its immediate bedside applicability. Because all six parameters are obtainable from clinical history and routine laboratory tests (blood count and CRP) within minutes and at no additional cost, the score can be applied in any emergency department, including hospitals without on-site catheterization facilities, and it guides triage toward the appropriate diagnostic pathway, CMR or EMB for suspected myocarditis vs. coronary angiography for ACS, particularly when the two conditions are difficult to separate, such as in chest-pain patients who are neither clearly young nor old. Notably, the high-specificity cutoff (≥7 points; specificity >99.9%) identifies patients in whom acute myocarditis is highly probable, a scenario in which inter-hospital transfer for coronary angiography, with its 1%–2% complication risk, radiation burden, and costs, could potentially be avoided or de-prioritized in hemodynamically stable patients. Its retrospective, single-center derivation and modest validation cohort (*n* = 90) nevertheless mandate prospective, multicentric confirmation, ideally in rural and developing-country settings where its triage value would be greatest.

**Table 2:** The SAlzburg MYocarditis (SAMY) score: components, points, diagnostic cutoffs and performance.

Parameter	Level	Points
Age	≤35 years	+5
	36–50 years	+4
	51–65 years	+2
	≥66 years	0
CRP	≤80.0 mg/L	0
	80.1–150.0 mg/L	+4
	150.1–250.0 mg/L	+2
	≥250.1 mg/L	0
Leukocyte count	≤9.0 G/L	+3
	9.1–12.5 G/L	+1
	12.6–15.5 G/L	−2
	≥15.6 G/L	0
Infection within 4 weeks	yes	+2
	no	0
Arterial hypertension	yes	−2
	no	0
Hyperlipidemia	yes	−2
	no	0

Diagnostic cutoffs and performance	
Cutoff #1 (≥4 points)	Sensitivity 90.3% Specificity 93.1% · PPV 87.5% NPV 94.7%
Cutoff #2 (≥7 points)	Sensitivity 73.1% Specificity >99.9% PPV >99.9% NPV 87.4%
Validation cohort (*n* = 90)	AUC 0.935; adequate calibration (retrospective, single-centre — prospective validation required)

AUC, area under the curve; CRP, C-reactive protein; NPV, negative predictive value; PPV, positive predictive value; SAMY, SAlzburg MYocarditis; Sens, sensitivity; Spec, specificity. Adapted from “Score sheet of the proposed clinical score for the prediction of myocarditis that includes the two calculated cutoffs (#1: ≥ 4, Sens.: 90.3%, Spec.: 93.1%, PPV: 87.5%, NPV 94.7%; 46.3% predicted probability for myocarditis; #2: ≥ 7, Sens.: 73.1%, Spec.: > 99.9%, PPV: > 99.9%, NPV 87.4%; 92.9% predicted probability for myocarditis)” by Moritz Mirna, Lukas Schmutzler, Albert Topf, Brigitte Sipos, Lukas Hehenwarter, Uta C. Hoppe and Michael Lichtenauer, licensed under CC BY 4.0.

### 5.2 Integrated multimodal algorithm

The algorithm proposed here does not replace guideline-directed pathways, which remain the standard of care. Rather, it synthesizes current ESC recommendations on ACS, myocarditis/pericarditis and SICM into a single operational sequence, integrating emerging biomarkers and the SAMY score at the points where guidelines leave room for clinical judgment, particularly in settings without immediate access to invasive coronary assessment. ECG and the first troponin measurement are performed immediately and in parallel, as shown in Figure 2.

**Figure 2:**
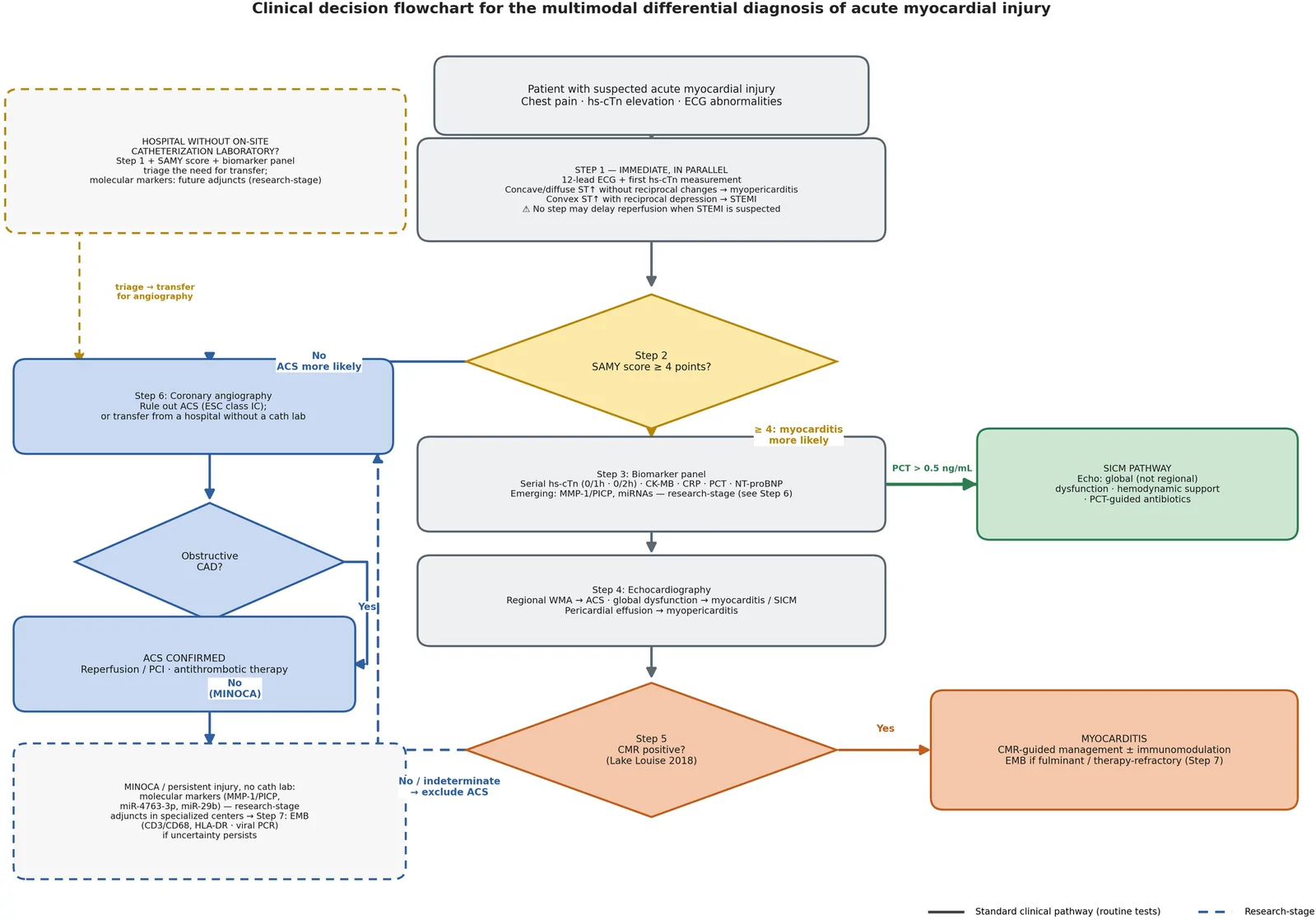
Clinical decision flowchart for the multimodal differential diagnosis of acute myocardial injury. ACS, acute coronary syndrome; CAD, coronary artery disease; CK-MB, creatine kinase-MB; CMR, cardiac magnetic resonance; CRP, C-reactive protein; ECG, electrocardiogram; EMB, endomyocardial biopsy; ESC, European society of cardiology; HLA-DR, human leukocyte antigen-DR; hs-ctn, high-sensitivity cardiac troponin; MMP-1, matrix metalloproteinase-1; MINOCA, myocardial infarction with non-obstructive coronary arteries; miRNA, microRNA; NT-proBNP, N-terminal pro-B-type natriuretic peptide; PCI, percutaneous coronary intervention; PCR, polymerase chain reaction; PCT, procalcitonin; PICP, procollagen type I carboxy-terminal propeptide; SAMY, SAlzburg MYocarditis; SICM, sepsis-induced cardiomyopathy; STEMI, ST-elevation myocardial infarction; WMA, wall motion abnormality.

Step 1: Immediately upon presentation, obtain a 12-lead ECG in parallel with the first hs-cTn measurement. Concave ST-segment elevation without reciprocal changes, particularly when diffuse across territories, favors myopericarditis/myocarditis over STEMI (convex elevation with reciprocal depression) (83). No subsequent step may delay reperfusion when STEMI is suspected.

Step 2: Assess clinical probability using the SAMY score or another validated tool, integrating age, recent infection, cardiovascular risk factors, CRP and leukocyte count.

Step 3: Biomarker panel including hs-cTn (with serial kinetics), CRP, PCT, and NT-proBNP, which is all routinely available in the emergency department. The pattern of cTn elevation (gradual vs. steep rise), inflammatory marker profiles, and biomarker ratios inform etiological probability. Emerging molecular markers, including MMP-1/PICP and circulating microRNAs such as miR-4763-3p, miR-29b and miR-155, are currently restricted to specialized or research settings and are therefore not yet incorporated into routine point-of-care algorithms.

Step 4: Echocardiography. Regional wall motion abnormalities favor ACS, whereas global dysfunction suggests myocarditis or SICM. The presence of pericardial effusion, particularly when combined with a pericarditic clinical phenotype, strongly supports myopericarditis rather than STEMI.

Step 5: Use advanced imaging with CMR and the updated Lake Louise Criteria (2018). Myocarditis typically shows T2-based edema, T1-based injury or inflammation (such as native T1 elevation and extracellular volume expansion), and specific late gadolinium enhancement patterns. In ACS, you will see subendocardial or transmural late enhancement that matches coronary territories (14, 84).

Step 6: Coronary angiography when ACS probability remains high despite a non-diagnostic workup (or, in hospitals without on-site catheterization facilities, after bedside triage to decide on transfer). When angiography reveals no obstructive coronary disease in the setting of acute myocardial injury, evaluation for myocarditis, vasospastic disorders or spontaneous coronary artery dissection is warranted. In this setting, molecular markers (MMP-1/PICP, microRNAs) may serve as research-stage adjuncts in specialized centers.

Step 7: Consider EMB in patients with unstable blood flow, significant arrhythmias, or when the diagnosis is unclear and will affect treatment. Immunohistochemical staining and viral genome analysis (PCR, *in-situ* hybridization) can confirm the exact cause (1).

A distinctive strength of the biomarker-based approach proposed here is its applicability where invasive coronary assessment is not immediately available. Among the markers reviewed in Section 4, hs-cTn, CRP, PCT and natriuretic peptides are standardized and already actionable in any emergency department, and the SAMY score converts these into a bedside probability estimate that can guide transfer decisions. By contrast, miRNA panels and the MMP-1/PICP axis are not yet ready for this purpose. The evidence derives mainly from small, retrospective, single-center cohorts, and neither internationally standardized assays nor validated cut-off values exist. Their clinically meaningful niche would be exactly this resource-limited setting, as non-invasive adjuncts that refine the myocarditis-vs.-ACS probability before transfer, but prospective multicentric validation, ideally in rural and developing-country cohorts, is a prerequisite. Until then, molecular markers remain confined to specialized centers and research use, and must not postpone guideline-recommended angiography and reperfusion when ACS cannot be excluded.

## 6 Conclusions and future perspectives

Diagnosing myocarditis, ACS, and SICM is often difficult and usually needs several tests. Hs-cTn is the main biomarker for all three, and its specific patterns, together with other biomarkers, help doctors begin to distinguish the causes. Tools such as the SAMY score also help doctors quickly assess risk at the bedside and guide patients to the right diagnostic steps.

Emerging biomarkers, particularly circulating miRNAs and extracellular matrix remodeling markers (MMP-1, PICP), demonstrate promising specificity for distinguishing inflammatory from ischemic myocardial injury. Their translation to routine clinical practice, however, faces substantial practical barriers that extend beyond analytical validation. Turnaround time is a decisive constraint in the emergency setting. Current miRNA quantification relies on RNA extraction followed by reverse transcription quantitative PCR or sequencing, a workflow requiring several hours to days and specialized laboratory infrastructure, which stands in sharp contrast to the 0/1 h and 0/2 h hs-cTn algorithms that define contemporary chest-pain triage. Pre-analytical factors further complicate bedside application, as miRNA measurements are sensitive to specimen type (serum vs. plasma), hemolysis, and storage conditions, and no universally accepted reference materials or calibrators currently exist. Assay standardization remains lacking for both miRNAs and the MMP-1/PICP axis. Inter-platform variability, absence of internationally validated decision thresholds, and unresolved harmonization across laboratories preclude result comparability. In addition, regulatory approval for cardiovascular diagnostic use, reagent cost, and the need for trained personnel limit deployment outside specialized centers. Accordingly, the realistic near-term role of these markers is as adjunctive tools within research settings, and multi-biomarker panels incorporating established and novel markers may ultimately provide the accuracy needed for point-of-care diagnostic algorithms only after these operational hurdles are addressed.
